# XSiteTraj: A cross-site user trajectory dataset

**DOI:** 10.1016/j.dib.2023.109783

**Published:** 2023-11-07

**Authors:** Jiazheng Fu, Yongjun Li

**Affiliations:** School of Computer, Northwestern Polytechnical University, Xi'an, Shaanxi 710072, PR China

**Keywords:** Social networks, Check-in data, User identification, Match user accounts

## Abstract

With the development of mobile networks, social networking plays an increasingly important role in people's daily life. User identification, which aims to match user cross-site accounts, has been becoming an important issue for user supervision and recommendation system design in social networks.

At present, many different user identification methods have emerged, such as DPLink, HFUL, etc. However, compared with the continuous development of user identification methods, the open-source work of datasets is very slow, and the datasets of most of the work are not public. The shortage of datasets has greatly hindered the development of this research field. At present, the academic urgently needs a large-scale social network user linkage dataset.

In this paper, we publicize a new social network user linkage dataset, XSiteTraj v1.0 [2]. This dataset has good spatio-temporal coverage, including more than 27,000 users and more than one million check-in records from all over the world crawled from Facebook, Foursquare, and Twitter. Our dataset labels the identical users from different social websites, and each check-in record includes a timestamp, point of interest (PoI), and latitude and longitude of PoI. Through our dataset, we can conduct research on user behaviour habits and apply the dataset to the experiments and evaluation of social network user identification and other algorithms.

Specifications Table

**Table:** 

Subject	Computer Sciences / Information Systems
Specific subject area	The dataset can be used for cross-site user identification, that is, to extract the identical users across different social network platforms using the original social network user check-in data, which is an important issue in social network analysis.
Data format	Raw data in .csv format.
Type of data	.csv file containing user trajectories.
Data collection	We use python's scrapy framework to write a distributed crawler to crawl user check-in information on Facebook, Foursquare, and Twitter. By extracting the information of the website page, the PoI, latitude and longitude, and time stamp of the check-in can be obtained, and the identical user on different social platforms can also be obtained through the binding information of the user's third-party account. These user data are desensitized and sorted into csv files.
Data source location	Crawl check-in trajectory data from Facebook, Twitter, and Foursquare social platform users around the world.
Data accessibility	Repository name: Zenodo Data identification number: 10.5281/zenodo.10035739 Direct URL to data: https://doi.org/10.5281/zenodo.10035739
Related research article	[1] Y. Zhang, Y. Li, W. Ji, A Trajectory-Based User Movement Pattern Similarity Measure for User Identification, IEEE Trans Netw Sci Eng. 10(6): 3834-3845, 2023. https://doi.org/10.1109/TNSE.2023.3274516

## 1 Value of the data

• The mining of check-in information on social networks can bring huge economic and social benefits to the society. Only by linking two users across different social networks can we better dig out the potential behavior habits of users, to implement user supervision, recommendation systems and other applications.
• Because there is very little association information between different social network users, that is, users will not be associated to third-party accounts, makes it is difficult to obtain a dataset for social network user identification tasks. Our dataset includes association information of users of different social sites, which can reduce the research cost of other researchers and promote the development of the research field.
• Social network analysis researchers can use this dataset for cross-site user identification. They can also only use single platform dataset, or combine datasets based on user association information to study trajectory-user linkage, PoI recommendation and other issues.

## 2 Data description

**Fig. 1:**
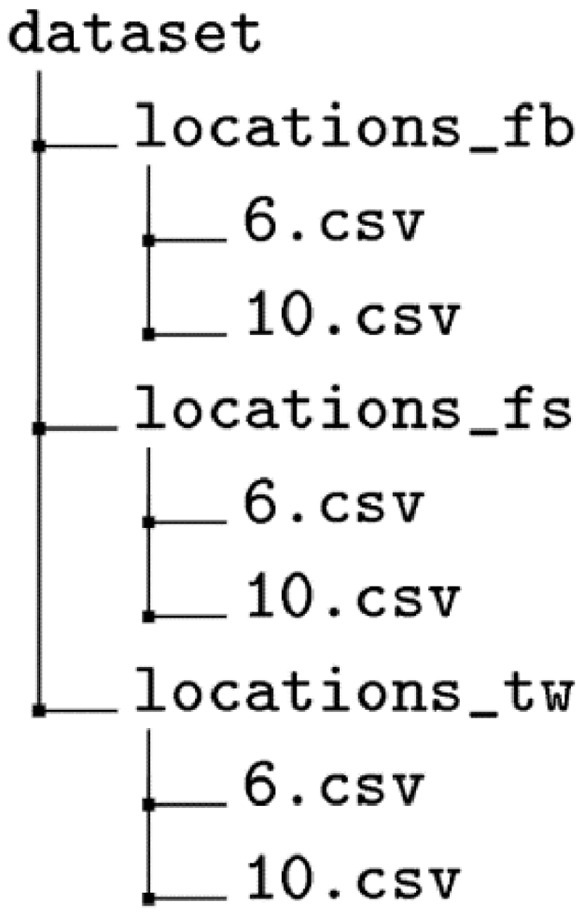
Illustration of dataset directory structure.

We divide the trajectory dataset of each social network into multiple files according to different users. In Fig. 1 we give a simplified illustration of the dataset directory structure. The name of each file is the user id, and the data under different social networks are stored in different folders. Trajectories generated by the identical user are stored in files with the same name in different folders. The file stores all check-in records of a certain user in csv format, and all data has been desensitized to protect the sensitive information of the user.

The format of the dataset is shown in Table 1. Each check-in contains timestamp, longitude, latitude, and corresponding PoI. The time is all in the user's local time zone, the latitude and longitude are in floating-point format, and the PoI is the location crawled from the user's check-in information.

**Table 1:** Dataset format.

Column	Format	Description
PoI	String	PoI of the trajectory record
Time	String	Timestamp of the trajectory record
(Longitude, Latitude)	Float	Latitude and longitude of the PoI

**Fig. 2:**
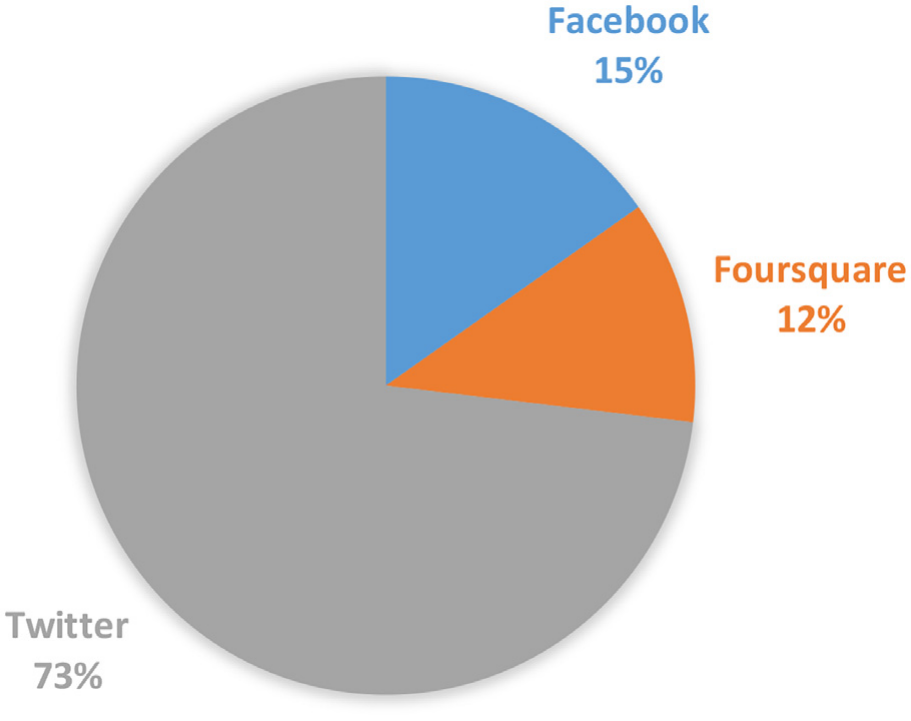
Data count from each social media.

Table 2 shows the basic information of the dataset, including the number of users, the number of check-in records and the total number of PoIs in the dataset. Fig. 2 shows the number of check-in records on different social media. Twitter has the largest count of data, accounting for 73%, followed by Facebook and Foursquare, accounting for 15% and 12% respectively.

**Table 2:** Dataset statistics.

Dataset	User Count	Check-in Records	PoI Count
Facebook	7158	312,574	17,090
Foursquare	8593	240,081	143,252
Twitter	11,856	1,502,428	65,190

In Table 3, we show the check-in records of the user with id 31 on three platforms in August 2014. It can be seen that PoI is the location where the user locates when checking in. On Foursquare, it will be specific to a certain store, while on other platforms, it will be in a region or city. The latitude and longitude are the geographic coordinates of the PoI on the map, and the conversion between the two is done by the Bing Map API.

**Table 3:** Check-in records of user(id=31) in August 2014.

Dataset	Time	PoI	(Longitude, Latitude)
Facebook	2014/8/17 18:09	Diamond Beach, NJ, United States	(38.95757675, −74.84957886)
	2014/8/16 16:37	Diamond Beach, NJ, United States	(38.95757675, −74.84957886)
	2014/8/15 9:27	New York, NY, United States	(40.75325012, −74.00380707)
Foursquare	2014/8/25 7:13	The Little Daisy Bake Shop, 622 Valley Rd, Montclair	(40.84196782, −74.20823093)
Twitter	2014/8/25 7:56	Greenpoint, Brooklyn	(40.72709274, −73.94673157)
	2014/8/25 8:08	Philadelphia, PA	(39.95106125, −75.1656189)
	2014/8/24 21:24	San Francisco, CA	(37.78007889, −122.4201584)
	2014/8/17 20:04	Manhattan, NY	(40.75325012, −74.00380707)
	2014/8/13 16:36	San Francisco, CA	(37.78007889, −122.4201584)
	2014/8/6 7:51	New York, NY	(40.71304703, −74.00723267)
	2014/8/1 22:57	San Francisco, CA	(37.78007889, −122.4201584)

**Fig. 3:**
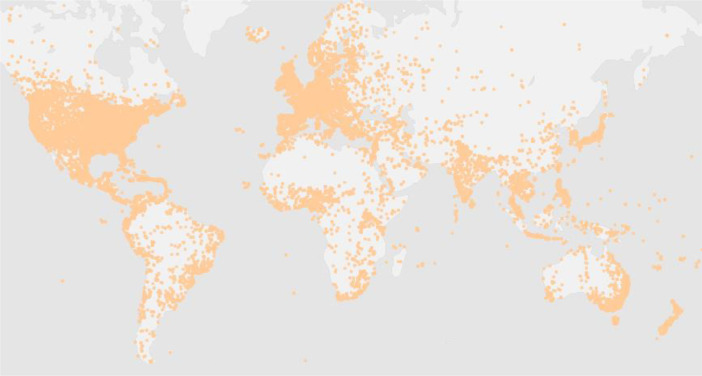
Spatial coverage (The Whole World).

In terms of the distribution of trajectories, as shown in Fig. 3, we visualize the recording points of the user's check-in. Check-in records are distributed all over the world, mainly in North America and Europe.

## 3 Experimental design, materials and methods

**Fig. 4:**
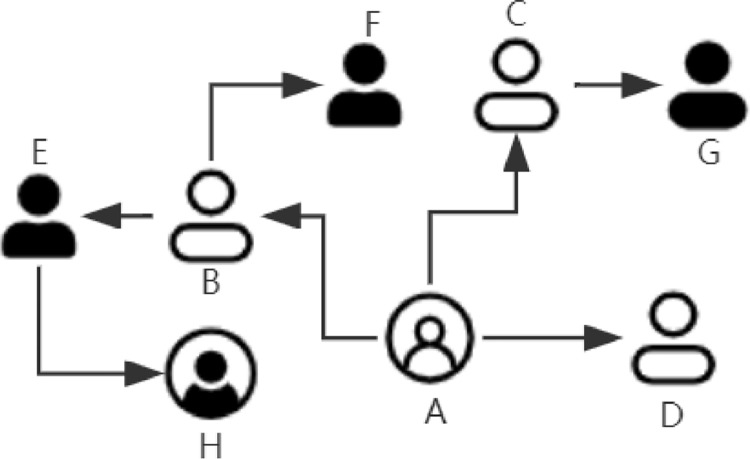
Demonstration of users’ relationship information.

The dataset is crawled from Facebook, Foursquare, and Twitter. In order to obtain a part of the user set in the social network, we select some seed users and perform breadth-first traversal on the user relationship network. Fig. 4 shows an example of a user relationship network. Different user icons represent users traversed at different levels of breadth-first traversal, and arrows represent the following relationship among users. In this example, user A is a seed user, through it we can get neighbors {B, C, D}, second-order neighbors {E, F, G}, and third-order neighbors {H}. In view of the large scale of social network users, we use Python's Scrapy framework to implement crawlers and deploy them on multiple servers for distributed crawling. When crawling, we need to simulate an HTTP request to obtain the corresponding web page response, and then extract and analyze the response body. For each user traversed, we use xpath to extract their tweets, check-in, and other information, and then store them in the database.

After large-scale crawling of the network, we can finally obtain the check-in information of some users in the corresponding social platform. Because each user may bind accounts of other websites in their own account information, we can use this to obtain the identical users in different social networks. If the website does not provide latitude and longitude or PoI, we can use the Bing Map API to convert PoI and latitude and longitude. The specific crawling strategies of different social networks are as follows.

For Twitter, we leverage its developer API [3]. First, we can get following information through the Twitter Follower API, and then get the user's tweet information through the Twitter Timeline API.API responses are all in JSON format, which can be analyzed directly using Python. If there is a location in the user's tweet, we extract the timestamp, latitude and longitude, PoI, and thus find a check-in record. Twitter's official website describes the Twitter's rate limiting measures, and we need to perform account switching and rate limiting based on this.

For Facebook, we scan the user's homepage and get the corresponding HTML response. The breadth-first traversal of the network can be performed through the user's friends and fans list, and the check-in record can be obtained by scanning the published posts [4]. Based on the association information of other social networks added to the personal information, we can find the identical user in different websites.

For Foursquare, through our analysis of the website structure, we find the underlying API interface for web page loading, so that we only need to simulate the HTTP request for the API to get the user's following page and check-in page. After obtaining the HTML response, we use the xpath expression to extract information from the page to obtain the PoI, latitude, and longitude of the check-in. Foursquare supports third-party login. If the user binds a third-party account, the user's personal information column will have a Twitter or Facebook icon, so that we can extract account-related information by analyzing the user's personal information page.

## Limitations

Compared with the datasets based on GPS trajectories, the check-in dataset is very sparse, which will make it difficult to analyse user's movement pattern. In addition, because user account association information is difficult to obtain, there are few labelled users in the dataset. That is, for a certain unlabelled user, we do not know who the identical user on other platforms is. Existing approaches cannot use this part of data in model training, which hinders the improvement of the model effect.

## Ethics statement

We confirm that:

a) Participant data has been fully anonymized.
b) The platform(s)’ data redistribution policies were complied with.
c) All personal privacy information in the public dataset has been deleted. It only contains IDs that are irrelevant to the user, latitude and longitude, and PoI, and ensures that individuals cannot be identified through check-in record sequences.

## CRediT authorship contribution statement

**Jiazheng Fu:** Methodology, Data curation, Investigation, Writing – review & editing. **Yongjun Li:** Supervision, Conceptualization, Resources.

## Data Availability

XSiteTraj: A Cross-site User Trajectory Dataset for User Linkage (Original data) (Zenodo)

## References

[1] Zhang Y., Li Y., Ji W. (2023). A trajectory-based user movement pattern similarity measure for user identification. IEEE Trans. Netw. Sci. Eng..

[2] Fu J., Li Y. XSiteTraj: a cross-site user trajectory dataset for user linkage, Zenodo, 2023.

[3] S.S. Sohail, M.M. Khan, M. Arsalan, A. Khan, J. Siddiqui, S.H. Hasan, M.A. Alam, Crawling Twitter data through API: a technical/legal perspective, ArXiv Preprint ArXiv:2105.10724. (2021).

[4] Catanese S.A., De Meo P., Ferrara E., Fiumara G., Provetti A. (2011). Proceedings of the International Conference on Web Intelligence, Mining and Semantics.

